# Uncommon Presentation of Lepromatous Leprosy in a Nonendemic Setting: A Case Report

**DOI:** 10.1155/carm/8232445

**Published:** 2025-09-12

**Authors:** Mhdia Elhadi Osman, Tareq Nafea Alharby, Yasser Alhabeeb, Ayman Elshenawy, Hala Ghazi Alreshidi, Saja Saleem Saja Saleem Alhayeti, Areej Alhumaidi Alshammari

**Affiliations:** ^1^Department of Clinical Pharmacy, College of Pharmacy, University of Ha'il, Ha'il 81442, Saudi Arabia; ^2^Department of Clinical Pharmacy, King Khalid Hospital, Hail Health Cluster, Ha'il, Saudi Arabia; ^3^Dermatology Department, King Khalid Hospital, Hail Health Cluster, Ha'il, Saudi Arabia; ^4^Internship College of Pharmacy, University of Ha'il, Ha'il, Saudi Arabia

**Keywords:** case report, granulomatous disorders, Hansen's disease, lepromatous leprosy, *Mycobacterium leprae*

## Abstract

Leprosy, caused by *Mycobacterium leprae*, remains a significant public health concern in certain endemic regions, but it is rarely encountered in nonendemic areas, posing diagnostic challenges. This case report discusses an unusual presentation of lepromatous leprosy in a patient residing in Saudi Arabia with no history of travel to endemic regions. The patient's clinical features were atypical, lacking the classic neurological involvement and sensation loss commonly associated with leprosy. Definitive diagnosis was achieved through histopathological examination revealing diffuse histiocytic infiltrates, a grenz zone, and acid-fast bacilli confirmed by modified Ziehl–Neelsen staining. The patient responded well to multidrug therapy according to WHO guidelines. This case highlights the importance of maintaining high clinical suspicion, utilizing appropriate diagnostic techniques, and understanding the epidemiological complexities of leprosy in low-prevalence settings. Strengthening awareness and surveillance is essential to prevent underdiagnosis and curb transmission in nonendemic regions.

## 1. Introduction

Leprosy or Hansen's disease is caused by *Mycobacterium leprae*, an intracellular, acid-fast bacillus (AFB). *M. leprae* invades small sensory and autonomic nerve fibers in the skin, resulting in damage that impairs hair growth, sebaceous gland secretion, sweating, and skin pigmentation. The bacteria are present in the dermal papilla and outer root sheath of both active and resting hair follicles [1, 2]. The bacterium binds to these cells, leading to their invasion and subsequent demyelination. This process disrupts nerve signal transmission, resulting in sensory and motor deficits [3]. Another mechanism is Type 1 reactions (also called reversal reactions), which involve immune-mediated inflammation that enhances the cell-mediated immune response against *M. leprae* antigens, leading to inflammation and swelling of nerves. This immune response can cause acute nerve damage if not promptly managed. The second type, erythema nodosum leprosum (Type 2 reactions), is an immune complex-mediated reaction causing systemic inflammation, which can further damage nerves [4]. Leprosy is primarily transmitted through prolonged close contact with an untreated individual. It can also spread via respiratory droplets, such as those produced when an infected person coughs or sneezes, or through direct skin-to-skin contact [5].

Leprosy is classified into tuberculoid (TT), borderline TT (BT), mid-borderline (BB), borderline lepromatous (BL), and lepromatous (LL) [6]. TT presents as one or more spots raised, slightly reddish, numb plaques that may peel. BT is characterized by sharper lesions that are localized in one part of the body and contain fewer bacteria. The most severe type, LL, also features erythematous macules, but these lesions are larger and do not separate over time, often leading to hair loss in the eyebrows and eyelashes [7].

*Mycobacterium leprae* primarily targets the skin, eyes, nerves, and mucosal lining. It can change the person's features. Transmission also occurs via nasal secretion [8]. Leprosy affects the entire body, including the skin, with significant consequences. Recognizing symptoms (e.g., sensory loss predisposing patients to trauma, infections, and muscle atrophy) is critical for effective treatment [9, 10]. Studies indicate *M. leprae* proliferates near sebaceous glands but cannot survive temperatures above 35°C [7].

Following the introduction of multidrug therapy (MDT), global leprosy cases dropped significantly from over five million in the 1980s to 133,802 in 2021, with a prevalence of 16.9 per million. However, leprosy remains endemic in many developing regions, particularly in Southeast Asia, Africa, and the Americas, with India, Brazil, and Indonesia reporting the highest number of new cases, each exceeding 10,000 annually'. In Saudi Arabia, Assiri et al. reported 242 new cases of leprosy from 2003 to 2012, with 57% occurring in immigrants, and a 3:1 male-to-female prevalence ratio [11, 12].

Treatment of leprosy is standardized worldwide and is based on World Health Organization (WHO) recommendations issued in 1982 that include three key medications: rifampicin 600 mg oral once daily with a meal, dapsone 100 mg oral once daily for all forms of leprosy, and clofazimine 50 mg oral once daily for 12–18 months [13]. This MDT has significant drawbacks, such as drug toxicity and poor adherence, which impact the quality of life of these patients. Dapsone is associated with hemolytic anemia, exfoliative dermatitis, and hypersensitivity reactions. Furthermore, clofazimine can cause skin discoloration, which tends to localize in plaques and nodules on the face and extremities. In addition, daily rifampicin may cause serious drug–drug interactions, reduced glucocorticoid efficacy, hepatotoxicity, acute interstitial nephritis, and other systemic side effects [13, 14]. Regarding postexposure prophylaxis, individuals in close contact with the infected patient for more than 20 h per week for over 3 months should be initiated on rifampicin 600 mg orally once daily [15].

The primary challenge in leprosy is identifying the source of transmission and explaining the absence of traditional risk factors, such as exposure to armadillos or travel to endemic areas [16]. Early and accurate detection of new cases, particularly among children and high-risk males, is crucial to reducing disease burden and preventing disabilities [17]. To decrease the transmission, preventive measures such as giving a single dose of rifampicin to close contacts of infected individuals are recommended. This strategy not only protects exposed persons from contracting leprosy but also prevents the illness from spreading further. Posttreatment surveillance protocols are essential for detecting relapses or drug resistance [18].

## 2. Case Report

A 42-year-old non-Saudi male who came to King Khalid Hospital in the Hail region of Saudi Arabia, a nonsmoker with no significant past medical history, family history of similar conditions, or prior medication use. In addition, he reported no history of international travel.

### 2.1. Family History

The patient's family history was unremarkable, with no evidence of similar conditions, hereditary diseases, or chronic illnesses.

### 2.2. Current Disease Status

The presenting symptoms include hyperpigmented plaques and nodules that are oval and rounded in shape. These lesions were nonitchy, nontender, and nonpainful and had been present on the face, upper limbs, and lower limbs for several months. There was no loss of sensation in these lesions, although the patient experienced hair loss in the outer third of both eyebrows.

### 2.3. Differential Diagnosis

LL, granuloma annulare, or mucinosis.

### 2.4. Diagnosis

**Figure 1:**
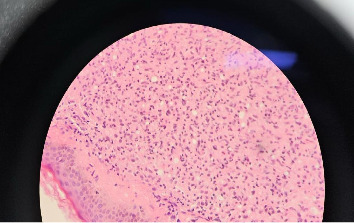
Diffuse histiocytic infiltrate with vague granulomatous reaction (H&E stain).

**Figure 2:**
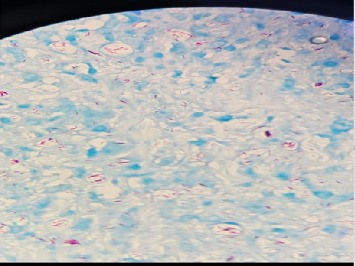
Modified ZN stain showed the lepromatous bacteria (*Mycobacterium lepra*).

The skin biopsy revealed abundant diffuse histiocytic infiltrate in the dermis covered by an unremarkable epidermal layer. A grenz zone of sparing was noted in the papillary dermis, and sparse lymphocytes were present. No nerve bundles were seen attached to the histiocytic infiltrate (see Figure 1). Modified Ziehl–Neelsen (ZN) staining (see Figure 2) demonstrated AFB, consistent with *Mycobacterium leprae*. The identification of lepra bacilli through this method confirmed the diagnosis of LL.

### 2.5. Laboratory Tests

Complete blood count with differential white blood cells, chemistry, liver function tests (LFTs), and thyroid function tests were within normal range (see Table 1).

### 2.6. Treatment Plan

The patient was initiated on the WHO-recommended MDT regimen: rifampicin 300 mg orally once monthly for 12 months, dapsone 100 mg orally once daily for 12 months, and clofazimine 100 mg orally once monthly, followed by 50 mg once daily for 12 months [18].

Monthly laboratory assessments included complete blood count, LFTs, comprehensive metabolic panel (chemistry), and glucose-6-phosphate dehydrogenase (G6PD) levels to ensure therapeutic safety and efficacy.

## 3. Discussion

This case of LL in a nonendemic setting highlights the diagnostic and epidemiological challenges associated with a rare disease in such regions. Given the low prevalence of leprosy in Saudi Arabia, the emergence of this case is particularly unusual [19].

Histopathological features, such as diffuse histiocytic infiltrates, a grenz zone, and AFB identified using modified ZN staining, were crucial in diagnosing LL [20]. The presence of a grenz zone, a hallmark feature seen under histological examination in LL, highlights the failure of the immune system to effectively limit the spread of *Mycobacterium leprae*. In addition, the absence of nerve bundle involvement observed in the biopsy differs from the usual pathological patterns seen in LL, suggesting that the degree of nerve tissue affinity might vary, particularly in the early or localized stages of the disease [21].

As for laboratory findings, the patient exhibited mildly elevated triglyceride levels along [22] with vitamin D deficiency [23]. While the association between these abnormalities and leprosy is not fully understood, they could point to coexisting metabolic or nutritional disturbances.

The patient's lack of travel history or known exposure to endemic regions complicates the transmission narrative, underscoring the role of subclinical or undiagnosed carriers in nonendemic areas. Notably, the absence of sensory loss in cutaneous lesions deviates from classic LL presentations, which often feature nerve involvement and anesthesia. This atypical presentation may delay diagnosis, as clinicians in nonendemic regions are less likely to suspect leprosy. The case also raises questions about transmission dynamics in nonendemic regions. As Saudi Arabia hosts a large immigrant population, latent infections or undiagnosed carriers may act as reservoirs, emphasizing the need for targeted screening in high-risk groups, as highlighted by Assiri et al. [24].

The patient's rapid response to WHO-recommended MDT aligns with global evidence, supporting MDT's efficacy in bacillary clearance and prevention of disability. However, long-term monitoring for drug toxicity (e.g., hepatotoxicity from rifampicin and hemolytic anemia from dapsone) remains critical. Despite progress in leprosy management, MDT remains the primary treatment and has effectively reduced disease incidence and transmission. However, rising antibiotic resistance has been reported, prompting the use of alternative antibiotics such as minocycline, clarithromycin, and fluoroquinolones for drug-resistant cases [25]'.

## 4. Conclusion

This study highlights the diagnostic challenges of leprosy in nonendemic areas, where atypical presentations and the absence of classic neurological symptoms can lead to delayed diagnosis and intervention.

Histopathological examination and specialized staining techniques were crucial in confirming the diagnosis.

The successful outcome with MDT as in the WHO guidelines and good adherence to this treatment further reinforce its global relevance, even in areas with low leprosy incidence.

Regarding future recommendations, policymakers in Saudi Arabia, including the Ministry of Health and the Ministry of Interior, should strengthen health screening protocols for individuals arriving from leprosy-endemic countries.

In conclusion, ongoing collaboration between health authorities, interministerial cooperation, and public education will enhance Saudi Arabia's capacity to manage imported infectious diseases such as leprosy.

Continually updating national guidelines and applying advanced surveillance strategies are critical to achieving global elimination goals while maintaining the Kingdom's low disease prevalence in an increasingly mobile world.

## Figures and Tables

**Table 1:** Laboratory profile in lepromatous leprosy: diagnostic findings and treatment monitoring parameters.

Laboratory test	Result	Normal range
Triglycerides	4.20 mmol/L	0–1.7 mmol/L
Cholesterol	5.06 mol/L	0–5.2 mmol/L
LDL	2.74	0–4.12 mmol/L
HDL	1.03	0–1.68 mmol/L
ALT	35.50	Normal
AST	28.50	Normal
Vitamin D	19.69	30–100
TSH	3.98	0.27–4
T3	4.99	3.1–6.8 pmol/L
T4	13.45	12–22 pmol/L
Calcium	2.35	2.12–2.52 mmol/L
Sodium	136.90	132–145 mmol/L
Potassium	4.07	3.2–5.1 mmol/L
Chloride	99.74	98–107 mmol/L
A1C	5.23	4.8–5.9
FBG	5.57	4.2–6.1 mmol/L
Total protein	80.70	66–87 q/L
Total bilirubin	9	0–21 μmol/L
Direct bilirubin	3	0–5
Albumin	45	Normal
Uric acid	357	202–416 μmol/L
GGT	36	5–61
Urea	4.20	2.76–8.07 mmol/L
Sr Cr	88	40–115 μmol/L

## Data Availability

All data are included in the manuscript.
